# Dietary Risk Assessment of *v-ATPase A* dsRNAs on Monarch Butterfly Larvae

**DOI:** 10.3389/fpls.2017.00242

**Published:** 2017-02-22

**Authors:** Huipeng Pan, Xiaowei Yang, Keith Bidne, Richard L. Hellmich, Blair D. Siegfried, Xuguo Zhou

**Affiliations:** ^1^Key Laboratory of Bio-Pesticide Innovation and Application of Guangdong Province, Department of Entomology, South China Agricultural UniversityGuangzhou, China; ^2^Department of Entomology, University of KentuckyLexington, KY, USA; ^3^Corn Insects and Crop Genetics Research Unit and Department of Entomology, Iowa State UniversityAmes, IA, USA; ^4^Department of Entomology and Nematology, University of FloridaGainesville, FL, USA

**Keywords:** RNAi transgenic plants, non-target organisms, *Danaus plexippus*, *Diabrotica virgifera virgifera*, life history traits, environmental risk assessment

## Abstract

By suppressing the expression of genes with essential biological functions, *in planta* RNAi can negatively affect the development and survival of target pests. As a part of a concerted effort to assess the risks of RNAi transgenic crops on non-target organisms, we developed an *in vivo* toxicity assay to examine the impacts of ingested dsRNAs incurred to the monarch butterfly, *Danaus plexippus* (L.), an iconic eco-indicator in North America. To create the worst case scenario, the full-length *v-ATPase A* cDNAs from the target pest, western corn rootworm, *Diabrotica virgifera virgifera*, and the non-target *D. plexippus* were respectively cloned. A 400 bp fragment with the highest sequence similarity between the two species was used as the template to synthesize dsRNAs for the subsequent dietary RNAi toxicity assay. Specifically, newly hatched neonates were provisioned with leaf disks surface-coated with *v-ATPase A* dsRNAs synthesized from *D. v. virgifera* and *D. plexippus*, respectively, a control dsRNA, β*-glucoruronidase*, from plants, and H_2_O. The endpoint measurements included gene expressions and life history traits. The 2283 bp *D. plexippus v-ATPase A* cDNA contains a 99 bp 5′-untranslated region, a 330 bp 3′-untranslated region, and an 1851 bp ORF encoding 617 amino acids. The temporal RNAi study did not detect any impact to *D. plexippus v-ATPase A* expression by the assay days and treatments. This was reflected in the phenotypic impacts of dietary RNAi, in which both survival rate and development time were not affected by the uptake of ingested dsRNAs. These combined results suggest that *D. plexippus* larvae are not susceptible to dietary RNAi, therefore, the impact of transgenic RNAi plants on this non-target organism is, likely, negligible.

## Introduction

RNA interference (RNAi) is a conserved mechanism that targets messenger RNA (mRNA) transcripts in a sequence specific manner and down regulates gene expression by interacting with small interfering RNAs (siRNAs). mRNAs can be silenced either through endogenous degradation via nuclease activity (e.g., RNAi pathway), or by translational repression (e.g., miRNA). The ability to modulate mRNA expression in insects provides an important genomic tool to elucidate gene functions (Bellés, 2010). More importantly, the ability to knock down genes of essential biological/physiological functions lays the foundation for the development of novel and sustainable pest control alternatives (Huvenne and Smagghe, 2010; Burand and Hunter, 2013; Xu et al., 2015). Dietary RNAi, which achieves sequence-specific gene silencing through voluntary feeding of double-stranded RNAs (dsRNAs), is a logical choice for pest control practices (Zhou et al., 2006; Baum et al., 2007; Zhu et al., 2011; Swevers and Smagghe, 2012; Scott et al., 2013).

The western corn rootworm, *Diabrotica virgifera virgifera* LeConte (Coleoptera: Chrysomelidae) is a serious pest of maize (*Zea mays* L.) throughout the US Corn Belt (Sappington et al., 2006). Rootworm management has integrated genetically engineered (GE) corn hybrids expressing *Bacillus thuringiensis* (Bt) toxins since early 2000. This GE technology has been adopted rapidly by growers but is currently threatened by the development of *Bt* resistance in the field and the lack of compatible control alternatives (Gassmann et al., 2011, 2014). RNAi can complement the existing *Bt* transgenic technology by offering a brand new mode of action (MOA) for the sustainable management of insect pests. *in planta* RNAi, delivering dsRNA through transgenic plants, has been pioneered in several insect pest species. Most recently, a GE event, MON 87411, which stacks one herbicide tolerance trait with two insect resistance traits, has been deregulated by the USDA's Animal and Plant Health Inspection Service (https://www.aphis.usda.gov/aphis/ourfocus/biotechnology/permits-notifications-petitions/petitions/petition-status). One of the GE traits designed to control *D. v. virgifera* involves a suppression cassette that targets *D. v. virgifera Snf7* gene (*DvSnf7*). Upon consumption, the plant-produced dsRNA in MON 87411 is recognized by *D. v. virgifera* RNAi machinery. The subsequent suppression of targeted *DvSnf7*, a housekeeping gene and an essential component of cellular machinery known as endosomal sorting complex required for transportation, leads to *D. v. virgifera* mortality (Bolognesi et al., 2012). Although technical difficulties and regulatory hurdles still exist (Lundgren and Duan, 2013; Casacuberta et al., 2015; Roberts et al., 2015), the commercialization of this new generation of GE crops is likely in the near future (Kupferschmidt, 2013; Levine et al., 2015).

One major environmental concern regarding the biosafety of GE crops on the environment is their potential effects on non-target organisms (NTO) (USEPA, 2013, 2014, 2016; EFSA, 2014), including the monarch butterfly, *Danaus plexippus* (L.) (Lepidoptera: Danainae). As the best known of all American butterflies, and an iconic eco-indicator in North America, *D. plexippus* is famous for its southward late summer/autumn migration from the United States and southern Canada to Mexico and coastal California, and northward return in spring, which occurs over the lifespan of three to four generations of the butterfly (Oberhauser and Solensky, 2004). In the past decade, *D. plexippus* has been served as a standard surrogate species to evaluate the impact of transgenic *Bt* crops on NTOs (USEPA, 2000; Hansen Jesse and Obrycki, 2000; Hellmich and Siegfried, 2001; Sears et al., 2001). Given that plant-expressed dsRNAs that targeted *D. v. virgifera v-ATPase subunits A* and *E* caused significant mortality in Colorado potato beetle, a non-target insect species (Baum et al., 2007), assessing the environmental risks of RNAi crops to *D. plexippus*, a non-target lepidopteran species incidentally inhabiting corn production systems during their annual migration, is warranted.

The current risk assessment framework for *Bt* crops has been recommended as a starting point to evaluate the potential hazards for RNAi crops (Auer and Frederick, 2009; Romeis et al., 2013; EFSA, 2014). Based on a tiered approach, we tested the overarching risk hypothesis that the stressor (e.g., arthropod-active dsRNA) does not adversely impact the non-target arthropods in the field (Romeis et al., 2008, 2011; EFSA, 2014). Tier I assessment is typically tested under the worst case scenario, which involves exposure to a maximum hazard dose (U.S. EPA suggest a margin of exposure factor of 10x) with purified active ingredients in artificial diets (USEPA, 1998; Romeis et al., 2008). Here, we hypothesized that *D. v. virgifera* active *v-ATPase A* dsRNA has no adverse impact on the non-target *D. plexippus*. To test this working hypothesis, we (1) cloned and sequenced *v-ATPase A* genes from *D. v. virgifera* and *D. plexippus*, respectively; (2) developed a dietary RNAi toxicity assay for *D. plexippus*; and (3) assessed the impacts of ingested *v-ATPase A* dsRNAs on the survival and development time of *D. plexippus*.

## Results

### Molecular cloning of *v-ATPase subunit A* gene

**Figure 1:**
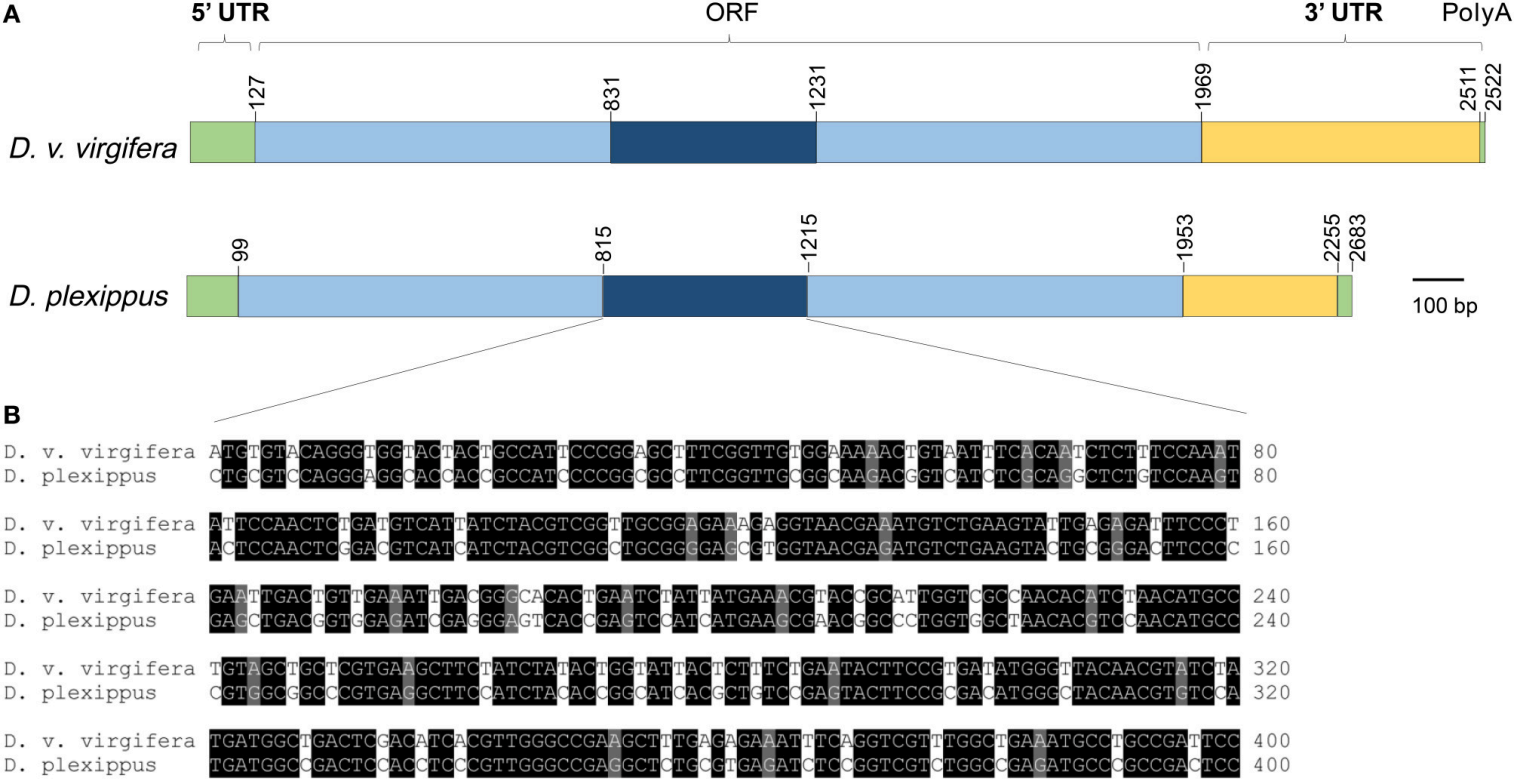
**Schematic comparison of *v-ATPase A* from insect pest *Diabrotica virgifera virgifera* and non-target *Danaus plexippus*. (A)** Schematic drawing of the primary structure of *v-ATPase A* cDNA from *D. plexippus* and *D. v. virgifera*. **(B)** The alignment of a highly conserved region within the ORFs of *v-ATPase A* from *D. plexippus* and *D. v. virgifera*. This 400 bp fragment, which has the highest sequence similarity among all the tested surrogate species, was selected as the template to synthesize insecticidal dsRNAs.

RT-PCR (reverse transcription polymerase chain reaction) and RACE (rapid amplification of cDNA ends) were used to amplify the entire coding sequence of the *v-ATPase A* from *D. plexippus. Danaus plexippus v-ATPase A* cDNA contains 2283 bp, including a 99 bp 5′-untranslated region, a 330 bp 3′-untranslated region, and an 1851 bp ORF encoding 617 amino acids (Figure 1A). *Diabrotica virgifera virgifera v-ATPase A* cDNA contains 2522 bp, including a 127 bp 5′-untranslated region, a 553 bp 3′-untranslated region, and an 1839 bp ORF that encodes 613 amino acids (Figure 1A). Pair-wise comparison of ORF indicates that the nucleotide sequence of *v-ATPase A* from *D. v. virgifera* shares 72.0% similarity with *D. plexippus* (Figure 1B, Figure S1). A 400 bp fragment with the highest sequence similarity (77.0%) between the two species was selected as the targeted region to synthesize the dsRNAs for the subsequent dietary RNAi toxicity assay (Figure 1B).

### Phylogenetic analysis

**Figure 2:**
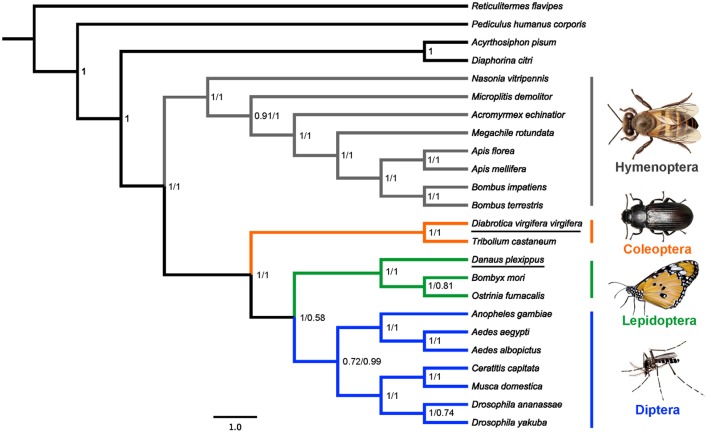
**Phylogenetic relationships of four holometabolous insect orders as inferred from *v-ATPase A* sequences**. Bayesian consensus tree obtained from analyses of the nucleotide datasets under GTR+I+G model. Values at nodes were Bayesian posterior probability (PP) using (from left to right) the datasets PCG (all codon positions with 1,842 nucleotides) and PCG12 (first and second codon positions with 1,228 nucleotides).

Bayesian analyses of two DNA sequence datasets provided identical topologies for the interordinal relationship of holometabolous insects (Figure 2). The monophyly of Holometabola (PP = 1.0), Hymenoptera (PP = 1.0), Coleoptera (PP = 1.0), Lepidoptera (PP = 1.0), and Diptera (PP = 0.72 and 0.99) were recovered, independently. Hymenoptera was the sister group to other three sampled holometabolous orders (PP = 1.0), and Coleoptera was the sister group to Lepidoptera and Diptera (PP = 1.0). This result is consistent with a recent phylogenomic analysis of insects based on 1,478 nuclear protein-coding genes (Misof et al., 2014).

### Bioinformatics analysis

The number of 19–25 nucleotide (nt) contiguous matches in the alignment of *v-ATPase A* sequences between *D. plexippus* and *D. v. virgifera* was analyzed by a customized bioinformatics tool. The result showed that there were no 19–25 nt contiguous sequence matches between the two insect species (Figure S1).

### Temporal profile of RNAi effects

**Figure 3:**
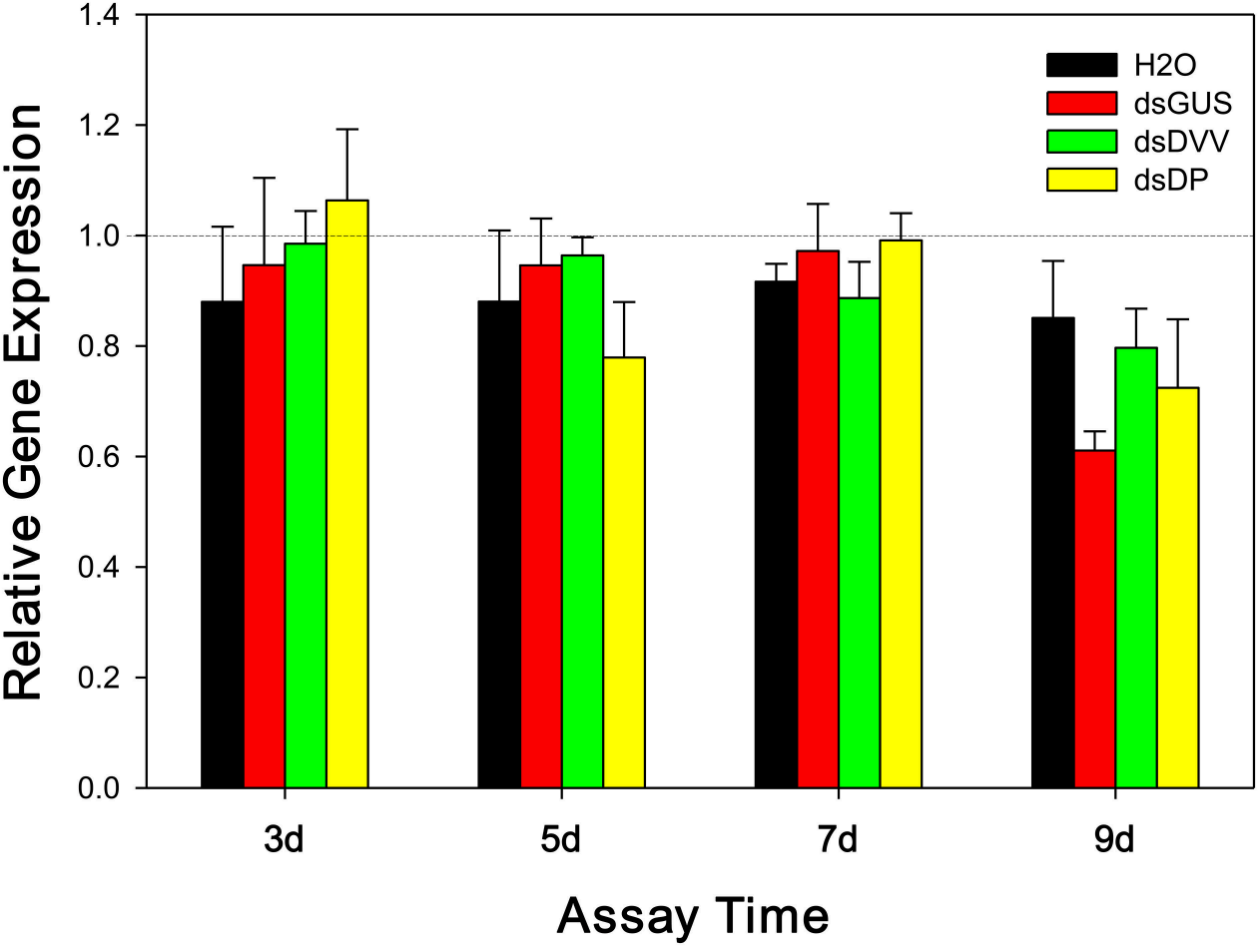
**Temporal expression of *D. plexippus v-ATPase* following the ingestion of dsDP**. The relative expression of *D. plexippus v-ATPase A* transcripts was normalized to reference genes, *EF1A* and *RP49*. The transcription level of *v-ATPase A* in newly emerged untreated larvae was set to 1, and the expression profiles in dsRNA-fed larvae were normalized to H_2_O, the vehicle control. Values are means + SE.

The expression of *D. plexippus v-ATPase A* was not affected by assay days [*F*_(3, 60)_ = 0.256, *P* = 0.857] and treatments [*F*_(2, 60)_ = 0.636, *P* = 0.533], and there was no interactions between these two factors [*F*_(6, 60)_ = 0.676, *P* = 0.669] (Figure 3).

### Dietary RNAi toxicity assay

**Figure 4:**
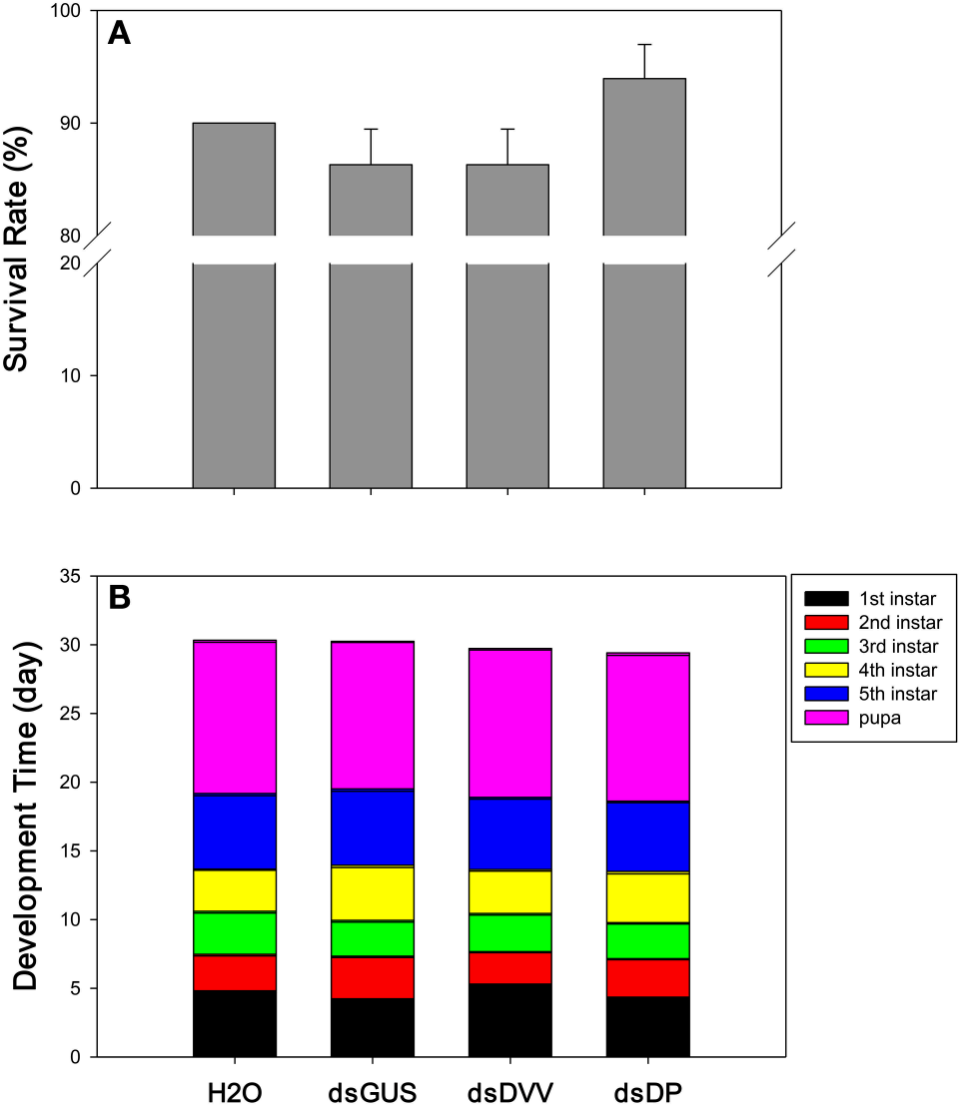
**Phenotypic impacts of dietary RNAi**. The endpoint measurements for *D. plexippus* dietary RNAi toxicity assay include both survival rate **(A)** and developmental time **(B)**. See Table 1 for detailed results. Values are means + SE.

There were no significant differences in the survival of *D. plexippus* larvae across treatments [*F*_(3, 8)_ = 1.823, *P* = 0.221] (Figure 4A). However, the development time of the 1st [*F*_(3, 113)_ = 9.498, *P* <0.001], 2nd [*F*_(3, 108)_ = 9.205, *P* <0.001], 3rd [*F*_(3, 103)_ = 3.735, *P* = 0.014], and 4th instar larvae [*F*_(3, 103)_ = 7.296, *P* <0.001] were all affected by the treatment; yet, the 5th instar larvae [*F*_(3, 103)_ = 1.676, *P* = 0.177] and pupal stage [*F*_(3, 103)_ = 1.520, *P* = 0.214] were unaffected by the treatment. Specifically, 1st instar larvae developed faster under the treatments of dsGUS and dsDP compared to the treatment of dsDVV. For the 2nd instars stage, *D. plexippus* had the fastest development time under the treatment of dsDVV, whereas it had the slowest development time under the treatment of dsGUS. For the 3rd instars stage, *D. plexippus* developed faster under the treatments of dsGUS and dsDP, whereas it had the slowest development time under the treatment of H_2_O. For the 4th instars stage, *D. plexippus* developed faster under the treatments of dsDVV and H_2_O compared to the treatments of dsGUS and dsDP (Table 1). Collectively, the development time from the 1st instar larvae to the adult was not affected by the treatments (Figure 4B).

**Table 1:** **The developmental time of *Danaus plexippus* following the ingestion of dsRNAs**.

**Life stage**		**Control**	**Treatment**		
		H_2_O (30)^*^	**dsGUS (33)**	**dsDVV (29)**	**dsDP (32)**
Larvae	1st Instar	4.67 ± 0.14b	4.07 ± 0.16c	5.13 ± 0.17a	4.22 ± 0.13c
	2nd Instar	2.54 ± 0.12bc	3.00 ± 0.10a	2.28 ± 0.08c	2.73 ± 0.08b
	3rd Instar	3.00 ± 0.14a	2.48 ± 0.13b	2.66 ± 0.12ab	2.52 ± 0.10b
	4th Instar	2.98 ± 0.09b	3.84 ± 0.17a	3.08 ± 0.12b	3.55 ± 0.18a
	5th Instar	5.33 ± 0.16a	5.38 ± 0.17a	5.12 ± 0.13a	5.00 ± 0.10a
Pupae		11.00 ± 0.15a	10.66 ± 0.09a	10.72 ± 0.11a	10.62 ± 0.18a

* *The number in the parenthesis denotes the quantity of neonates that were used in each treatment. One way ANOVA was used to compare the developmental time of D. plexippus under each treatment. LSD test declares the differences between means (P <0.05). Different letters indicate significant differences among treatments*.

## Discussion

### Creating the worst case scenario

In average, each neonate larvae ingested a single dose of approximately 16 μg of dsRNAs for the first 2 days of the toxicity assay, which is equivalent to an exposure of 1,600 times higher than the LC_50_ reported for *D. v. virgifera* larvae (Baum et al., 2007). As for *D. plexippus*, the most likely source of exposure to plant-expressed dsRNA is through the ingestion of maize pollen. Tan et al. (2016) reported that the maximum expected environmental concentration of *DvSnf7* dsRNA in MON 87411 pollen was 0.224 ng/g fresh weight (averaged expression level was 0.103, ranging from 0.056 to 0.224 ng/g fw) (Tan et al., 2016). In comparison, the estimated margin of exposure, i.e., the level of non-target exposure, in this study was at least 71,428 fold higher than the baseline expression level in the newly deregulated RNAi maize.

Besides the dose and exposure, the targeted 400 nt region of *v-ATPase A* accounted for the highest sequence similarity among targeted and non-target insect species, which, bioinformatically, created the best possible scenario for the non-target impacts. In a parallel study, we confirmed the toxicity of this 400 nt region of *v-ATPase A* to *D. v. virgifera* (Vélez et al., 2016). When fed with 1 μg/beetle dsDVV, rootworm adults reached 100% mortality in 9 assay days, while controls maintained approximately 10% mortality.

### Dietary RNAi toxicity assay

In this study, *D. plexippus* larvae were not responsive to the ingested *v-ATPase A* dsRNA, regardless of the sources, at both transcriptional and phenotypic levels. Terenius et al. (2011) summarized the challenges of RNAi in lepidopterans (Terenius et al., 2011). Targeted species, targeted tissue, delivery methods, dsRNA uptake, dsRNA degradation, and gene functions are factors contributing to RNAi efficiency. Among lepidopterans, RNAi is particularly successful in the family of Saturniidae (Terenius et al., 2011). In contrast, as a member of Danainae, *D. plexippus* appears to be rather refractory to the ingested dsRNAs. While injection has been used extensively for functional characterization studies (Roberts et al., 2015; Xu et al., 2015), oral exposure of dsRNA by dietary RNAi is the most relevant delivery method for the development of novel control alternatives, either through transgenic plants, baits, or formulations (Baum et al., 2007; Mao et al., 2007; Xu et al., 2015). Moreover, in Lepidoptera, RNAi is particularly sensitive for genes involved in immunity, whereas genes from protein binding group are refractory to silencing (Terenius et al., 2011). *v-ATPase* is a widespread membrane protein and an evolutionarily highly conserved enzyme that is found in all eukaryotic organisms (Nelson et al., 2000), the function of *v-ATPase* might contribute to its non-responsiveness to the ingested dsRNA in this study. As for the uptake, dsRNA can potentially be degraded prior to possible RNAi action (Whyard et al., 2009). Extracellular degradation of *in vitro* synthesized dsRNA was demonstrated in the saliva of tarnished plant bug, *Lygus lineolaris* (Allen and Walker, 2012) and in the gut and hemolymph of pea aphid, *Acyrthosiphon pisum* (Christiaens et al., 2014). *Danaus plexippus* have an active salivary gland which contains enzymes (e.g., dsRNase) to create an enzymatic barrier for dsRNA uptake.

Bioinformatically, the ability to silence genes by RNAi is based on 19–25 nt sequences identical to the target gene sequences (Bachman et al., 2013; Roberts et al., 2015). Based on their phylogenetic relatedness to *D. v. virgifera*, Bachman et al. (2013) carried out RNAi toxicity assays on an array of non-target insects, covering 10 families and 4 orders, using a *D. v. virgifera* active dsRNA, *DvSnf7*. This study suggested that a shared identical sequence of ≥21 nt in length (21 mer) was required for the efficacy against *D. v. virgifera*. Although *D. plexippus* and *D. v. virgifera* shared nearly 80% sequence similarity within the selected 400 nt region of *v-ATPase A*, the lack of 21 mer matches between target and non-target insect species suggest that impacts for *D. plexippus* through exposure to ingested *D. v. virgifera v-ATPase A* dsRNA is unlikely.

Although *D. plexippus* is apparently refractory to a systemic RNAi response, recent advances in the development of functional genomics tools provide promising alternatives for the study of gene functions in *D. plexippus*. By inducing irreversible DNA breaks, genome editing tools, including customized zinc-finger nucleases (ZFN), transcription activator-like effector nucleases (TALEN), or clustered regularly interspaced palindromic repeats-associated nuclease 9 (CRISPR-Cas9), can manipulate genomes at a specific locus (Harrison et al., 2014; Ochiai and Yamamoto, 2015). To circumvent the insensitivity to RNAi, CRISPR/Cas9 system has been adopted to produce genetic manipulations (knock in and knock out) in non-model organisms, including RNAi-recalcitrant Lepidoptera, such as *Bombyx mori* (Wang et al., 2013), *Spodoptera litura* (Bi et al., 2016; Zhu et al., 2016), *Papilio xuthus, P. machaon* (Li et al., 2015), and *D. plexippus* (Markert et al., 2016; Reppert et al., 2016). With the recent release of *D. plexippus* genome (Zhan et al., 2011), the CRISPR/Cas9 system will greatly facilitate the functional genomic research of this emerging insect model.

In summary, *D. plexippus* larvae seem to be refractory to a systemic response to RNAi and the effect of extraneously synthesized *v-ATPase A* dsRNAs on monarch butterfly is negligible. Although there were no significant differences in the survival and the overall development time, *D. plexippus* larvae indeed showed substantial variations in their growth time across treatments. Further studies evaluating the effects of multiple dsRNA exposures, different type of genes, sublethal impacts on individual life stages, potential off-target effects, and the environmental interactions between the RNAi crops and non-target *D. plexippus* will give us a holistic view of the impacts of *in planta* RNAi on this organism (Casacuberta et al., 2015; Devos et al., 2016). Moreover, the publication of negative results in risk assessments will help us document the susceptibility of specific organisms to this new GE trait and contribute to our overall understanding of what NTOs have the potential to be affected by the use of *in planta* RNAi in the environment (Roberts et al., 2015). This study provides a road map for future investigations on the risk assessment of RNAi crops. As a sequence-specific gene silencing technology that has a wide range of pest control potentials, additional surrogate species representing diverse ecosystem services should be tested in a similar fashion before moving RNAi-based gene silencing technology from the bench top to the table top.

## Materials and methods

### Insect and plant

#### Starting insect culture

Eggs of the monarch butterfly were collected from a common milkweed, *Asclepias syriaca*, near Ames, Iowa in spring and summer 2013. No specific permit was required for the field collection. Four to five generations of monarch adults were screened for presence of a protozoan parasite, *Ophryocystis elektroscirrha*, to eliminate it from the population. *Danaus plexippus* larvae were maintained in the laboratory on *A. syriaca* and tropical milkweed, *A. curassavica* at 25 ± 1°C and 16 L: 8 D photoperiodic regime at Iowa State University. Adults were fed with 15% sugar solution.

#### Working insect culture

Eggs from the starting insect cultures were shipped to the University of Kentucky for the subsequent risk assessment analyses. Upon arrival, eggs were kept at room temperature (25 ± 1°C) for several days, and allowed them to hatch. After newly hatched neonates (<24 h) consumed their egg shells, *D. plexippus* larvae were transferred to the petri dishes using a soft tip brush for the dietary RNAi toxicity assay. *Danaus plexippus* neonates were provisioned with leaf disks cut from honeyvine milkweed, *Cynanchum laeve* (*syn. Ampelamus albidus*). *D. plexippus* is a common American butterfly species in the United States. The permit to move live plant pests, noxious weeds, and soil were authorized by the United States Department of Agriculture Animal and Plant Health Inspection Service (Permit number: P526P-13-03521).

#### Plant materials

The honeyvine milkweed seed were collected at the North Farm, University of Kentucky. Milkweeds were grown in pots containing a mixture of vermiculite and organic fertilizer at 20–35°C, 60–100% RH in a nearby greenhouse. Leaf disc used in the dietary RNAi toxicity assay was from honeyvine milkweed.

### Molecular cloning and sequencing of *v-ATPase A*

Total RNA was extracted from one pair of *D. plexippus* adults using TRIzol® (Invitrogen, Carlsbad, CA) following manufacturer's instruction. First-strand cDNA was synthesized from 2.0 μg of total RNA using the M-MLV reverse transcription kit (Invitrogen, Carlsbad, CA) according to manufacturer's recommendations. Coding DNA Sequence (CDS) of *v-ATPase subunit A* (*v-ATPase A*) gene was extracted from the *D. plexippus* genome (Zhan et al., 2011). SMARTer® RACE cDNA Amplification Kit (TaKaRa Biotechnology (Dalian) Co., Ltd) was used to obtain the full length cDNA of *D. plexippus v-ATPase A* (Table 2). Amplicons of the expected size were purified and cloned into the pCR™ 4-TOPO® vector (Invitrogen, Carlsbad, CA) for sequencing confirmation. Cloning and sequence analyses of the full-length *v-ATPase A* transcripts were conducted on three independent batches of one pair of adults RNA, and at least six clones from each batch were verified by sequencing. The full length sequence of *D. v. virgifera v-ATPase A* was assembled from our transcriptome data (Eyun et al., 2014).

**Table 2:** **Primers used in this study**.

**Primers name**	**Sequence 5′−3′ (T7 = TAATACGACTCACTATAGGG)**
**PRIMERS USED For dsRNA Synthesis**	
dsDP-F	TAATACGACTCACTATAGGGAGATCGCTGTTCCCCTGC
dsDP-R	TAATACGACTCACTATAGGGAGAGCATCTCGGCCAGAC
dsDVV-F	TAATACGACTCACTATAGGGAGAGCTCTTTTCCCATGTGTAC
dsDVV-R	TAATACGACTCACTATAGGGAGAGCATTTCAGCCAAACG
dsGUS-F	TAATACGACTCACTATAGGGAGAGGGCGAACAGTTCCTGATTA
dsGUS-R	TAATACGACTCACTATAGGGAGAGGCACAGCACATCAAAGAGA
**PRIMERS USED FOR RT-qPCR**	
*v-ATPase A* RT-qPCR-F	AGGACGACTTCCTGCAACAGAACA
*v-ATPase A* RT-qPCR-R	TGTTCTTCAACATGCCCACCGTCT
*rp49* RT-qPCR-F	CCGGAAGGTGTTAGTCCACAAC
*rp49* RT-qPCR-R	CGGCGCAGTACTTCCTATTCTG
*EF1A* RT-qPCR-F	TGTCGCTTTCGTACCCATTT
*EF1A* RT-qPCR-R	CCTTCAGCCTTACCCTCTTTAC
**PRIMERS USED FOR GENE CLONING**	
DP *v-ATPase A* 5′RACE R	AGCATGGTGTACTGGTTCTTCTCTCCGTCGAA
DP *v-ATPase A* 3′RACE F	GGATGTGGTGCTGGAGACGGAGTT

### Phylogenetic analysis

*v-ATPase A* sequences from 24 insect species, including 20 holometabolous insects and four outgroup species from the orders Blattodea, Psocodea, and Hemiptera, were included in the phylogenetic analyses. The holometabolous insects include eight Hymenoptera, two Coleoptera, three Lepidoptera, and seven Diptera (Table S1). Sequences were aligned based on codon-based multiple alignments using the MAFFT algorithm in the TranslatorX online platform (Abascal et al., 2010). Ambiguously aligned sites were removed from the protein alignment before back-translation to nucleotides using GBlocks in TranslatorX with default settings. Alignments were then checked and corrected manually in MEGA v6.0 (Tamura et al., 2013). Two datasets were used in the analysis: (1) PCG: all codon positions with 1,842 nucleotides; (2) PCG12: first and second codon positions with 1,228 nucleotides. The GTR+I+G model was the best-fit nucleotide substitution model for two datasets selected by jModelTest 2 under AIC, BIC, and AICc criteria (Darriba et al., 2012). MrBayes v3.2.3 with GTR+I+G model was used for the phylogenetic analyses (Ronquist et al., 2012). Two simultaneous runs of 10 million generations were conducted for the dataset, and trees were sampled every 1,000 generations, with the first 25% discarded as burn-in. Stationarity was considered to be reached when the average standard deviation of split frequencies was below 0.01.

### Dietary RNAi toxicity assay

All bioassays were carried out in clear plastic creamers (1 oz., Bio-Serv, NJ) with five individuals in each creamer. For the first 2 days, newly hatched neonates were fed on leaf disks (dia. 0.5 cm) surface-coated with dsRNAs or vehicle (H_2_O) *ad libitum*. Specifically, 4.0 μl ddH_2_O containing 5.0 μg/μl of dsRNA or ddH_2_O only were dispensed onto each of the two leaf-disks (dia. 0.5 cm). Treatments and controls included *v-ATPase A* dsRNAs derived from *D. v. virgifera* (dsDVV) and *D. plexippus* (dsDP), respectively, a control gene, β*-glucuronidase* dsRNA derived from plant (dsGUS), and a vehicle control, H_2_O. Five *D. plexippus* neonate larvae consumed a total of four leaf disks (80.0 μg of dsRNAs) in the first 2 days. To keep the leaf disk fresh, 3.0 ml of 2% agar was added to the bottom of each creamer. On day-3, each larva was relocated to individual creamer (1 oz.) with 2% agar at the bottom; ample milkweed leaves were provided to feed the *D. plexippus* for the duration of its life cycle. At 4th instar, each individual larva was transferred to a larger container (15 oz., Pioneer Plastics). The life history traits of each larva were recorded, including survival and development time. Mortality and development stage for each larva were checked twice per day (9:00 a.m., 9:00 p.m.). For each treatment, approximately 10 neonates were used and each treatment replicated 3 times. Assays were run at 25 ± 1°C and 50% RH, under a 16 L: 8 D photoperiodic regime. The procedures for target region selection, *in vitro* synthesis of dsRNAs, transcriptional validation of dietary RNAi toxicity assay, and the subsequent statistical analyses are as follows.

#### Target region selection and bioinformatics analysis

To create the worst case scenario, a 400 nt fragment of *v-ATPase A* with the highest sequence similarity among target insect pest, *D. v. virgifera*, and non-target surrogate species representing diverse ecological functions was selected as the target region to synthesize dsRNAs. These non-target insect species include pollinators, *Apis mellifera* and *D. plexippus*, soil microanthropoids, *Folsomia candida*, and *Sinella curviseta*, and predatory biological control agents, *Hippodamia convergens, Harmonia axyridis, Coleomegilla maculata*, and *Coccinella septempunctata*. Pairwise sequence alignment was conducted between *D. v. virgifera* and each of the surrogate species via MUSCLE (Darriba et al., 2012). An in-house Perl script was used to determine the number of 19–25 nt continuous matches within this 400 nt region between *D. v. virgifera* and *D. plexippus*. The script searches for any instances of N continuous positions where there are no gaps in any sequences in the alignment. Insecticidal activity of this 400 nt region of *v-ATPase A* was validated in a parallel study against rootworm adults (Vélez et al., 2016).

#### dsRNA synthesis

Table 2 listed specific primers designed to generate dsDP, dsDVV, and dsGUS. As a non-specific negative control, GUS was cloned into pBTA2 vector and PCR amplified into a 560 bp fragment containing T7 polymerase promoter region at the 5′ end. PCR amplifications were performed in 50.0 μl reactions containing 10.0 μl 5 × PCR Buffer (Mg^2+^ Plus), 1.0 μl dNTP mix (10 mM of each nucleotide), 5.0 μl of each primer (10 μM each), and 0.25 μl of GoTaq (5 u/μl) (Promega). The PCR parameters were as follows: one cycle of 94°C for 3 min; 35 cycles of 94°C for 30 s, 59°C for 45 s, and 72°C for 1 min; a final cycle of 72°C for 10 min. The PCR product was used as template to generate dsRNA with the T7 MEGAscript kit (Ambion, Austin, TX, USA) following the manufacturer's protocol. The synthesized dsRNAs were resuspended in nuclease-free H_2_O and quantified with a NanoDrop 2000c spectrophotometer and before stored at −20°C.

#### Temporal profile of RNAi effects

During dietary RNAi toxicity assay, *D. plexippus* larvae were collected at day-0, 3, 5, and 7. All samples were snap frozen with liquid nitrogen and then transferred to 1.5 ml microcentrifuge tubes for the long-term storage at −80°C. The expression profiles of *D. plexippus v-ATPase A* were examined using reverse transcriptase-quantitative polymerase chain reaction (RT-qPCR). Total RNA was extracted using TRIzol® (Invitrogen, Carlsbad, CA) following the manufacturer's instruction. First-strand cDNA was synthesized from 0.7 μg of total RNA using the M-MLV reverse transcription kit (Invitrogen, Carlsbad, CA) and a random N primer according to manufacturer's recommendations. Gene-specific primers (Table 2) were used in PCR reactions (15 μl) containing 5.25 μl of ddH_2_O, 7.5 μl of 2 × SYBR Green MasterMix (BIO-RAD Inc., Hercules, CA), 4 μM of each specific primer, and 1.0 μl of first-strand cDNA template. The RT-qPCR program included an initial denaturation for 3 min at 95°C, followed by 40 cycles of denaturation at 95°C for 10 s, annealing for 30 s at 55°C, and extension for 30 s at 72°C. For melting curve analysis, a dissociation step cycle (55°C for 10 s, and then 0.5°C for 10 s until 95°C) was added. The reactions were set up in 96-well format Microseal PCR plates (BIO-RAD Inc., Hercules, CA) in triplicates. The relative expression of *D. plexippus v-ATPase A* transcripts was normalized to reference genes, *elongation factor 1*α (*EF1A*) and *ribosomal protein 49* (*RP49*) (Pan et al., 2015). Relative quantification of *v-ATPase A* in different samples was calculated using the 2^−^^ΔΔ*Ct*^ method (Livak and Schmittgen, 2001). Three technical replicates and six biological replicates were used for each treatment.

#### Statistical analysis

One way ANOVA was used to compare the survival rate and development time of *D. plexippus* under each treatment. Two-way ANOVAs were used to compare the gene expression profiles of *D. plexippus v-ATPase A* under different treatments and assay days. Means were compared with LSD tests at *P* <0.05. SPSS version 21.0 (SPSS Inc., Chicago, IL, USA) was used for statistical analyses.

## Author contributions

XZ designed the experiment. HP, XY performed the experiment. KB, XZ contributed reagents/ materials. HP, XZ, RH, BS wrote the paper.

### Conflict of interest statement

The authors declare that the research was conducted in the absence of any commercial or financial relationships that could be construed as a potential conflict of interest.
