# Supplementary material for: Dietary Risk Assessment of v-ATPase A dsRNAs on Monarch Butterfly Larvae
Source: Front Plant Sci. 2017 Feb 22;8:242. doi: 10.3389/fpls.2017.00242 (PMC5319984; doi:10.3389/fpls.2017.00242)
Supplement: Supplementary file 1 [file DataSheet1.docx]

**Supplementary Material**


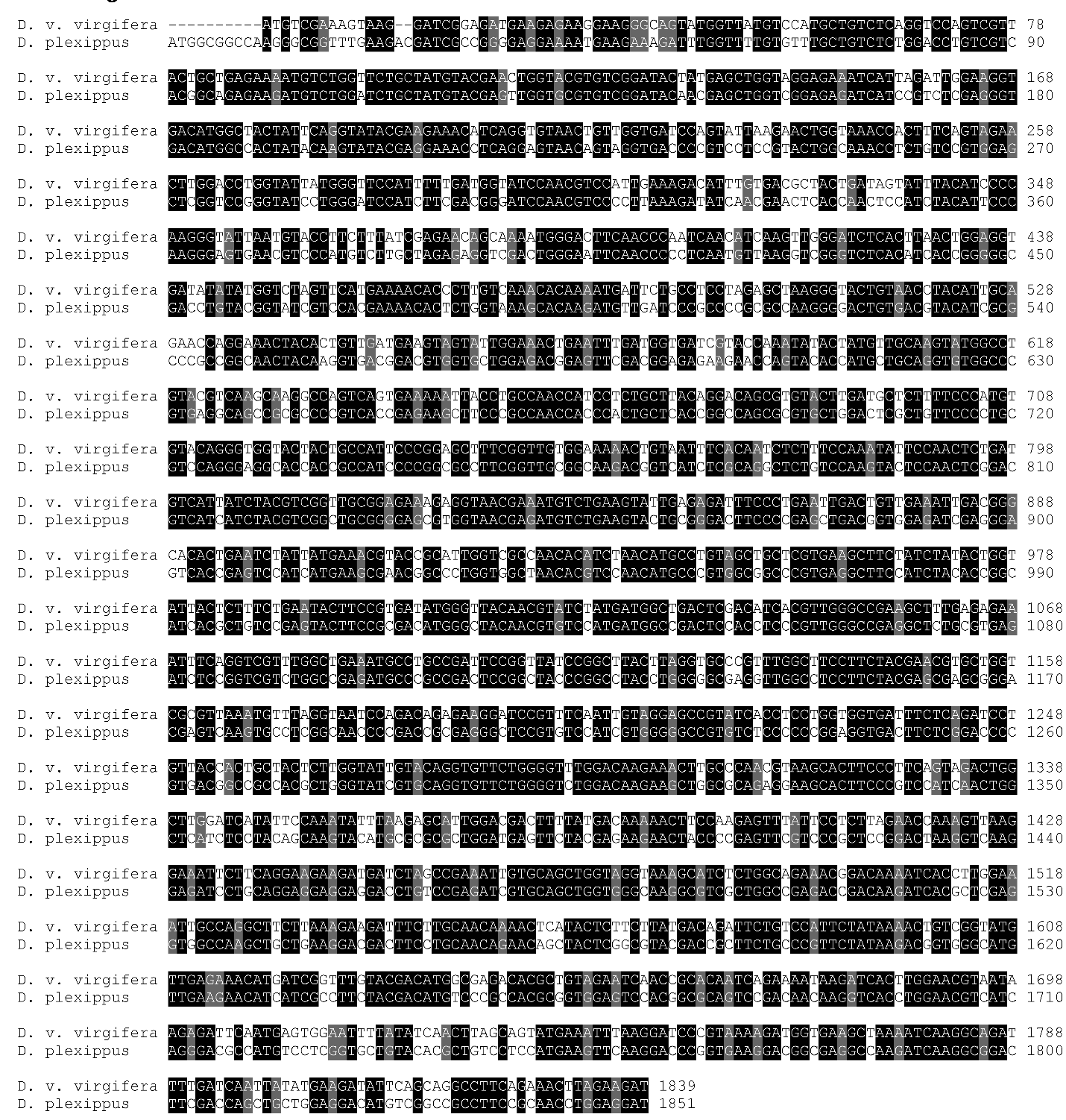


Figure S1

**Table S1. Insect information used for the phylogenetic analysis**

| **Order** | **Species** | **GenBank No.** |
| --- | --- | --- |
| **Hymenoptera** | *Nasonia vitripennis* | XM_008208806 |
|  | *Microplitis demolitor* | XM_008554975 |
|  | *Acromyrmex echinatior* | XM_011051974 |
|  | *Megachile rotundata* | XM_003699991 |
|  | *Apis florea* | XM_003690164 |
|  | *Apis mellifera* | XM_006567413 |
|  | *Bombus impatiens* | XM_003492267 |
|  | *Bombus terrestris* | XM_003397802 |
| **Coleoptera** | *Tribolium castaneum* | XM_971095 |
|  | *Diabrotica virgifera virgifera* | This study |
| **Lepidoptera** | *Danaus plexippus* | This study |
|  | *Bombyx mori* | NM_001098359 |
|  | *Ostrinia furnacalis* | FR727328 |
| **Diptera** | *Anopheles gambiae* | XM_003436363 |
|  | *Aedes aegypti* | AF008922 |
|  | *Aedes albopictus* | AY864912 |
|  | *Ceratitis capitata* | XM_004533325 |
|  | *Musca domestica* | XM_005179917 |
|  | *Drosophila ananassae* | XM_001962829 |
|  | *Drosophila yakuba* | XM_002088485 |
| **Out group** | *Acyrthosiphon pisum* | XM_008181186 |
|  | *Diaphorina citri* | XM_008471983 |
|  | *Reticulitermes flavipes* | KC569741 |
|  | *Pediculus humanus corporis* | XM_002426925 |
